# A new species of *Bolbelasmus* (Coleoptera, Bolboceratidae) from Guangdong Province, China

**DOI:** 10.3897/BDJ.12.e131664

**Published:** 2024-10-02

**Authors:** Weiqi Liao, Zhengwei Wu

**Affiliations:** 1 Guangdong Ocean University, Zhanjiang City, China Guangdong Ocean University Zhanjiang City China

**Keywords:** *Bolbelasmus*, new species, Guangdong, China

## Abstract

**Background:**

*Bolbelasmus* is a group of small to medium-sized beetles distributed in the Holarctic and Oriental Regions and in Central America. It includes two subgenera (*Bolbelasmus* and *Kolbeus*) and 32 species known in the world fauna with seven species recorded from China.

**New information:**

The new species, *Bolbelasmusguangdongensis* sp. nov., is described from south China and compared with *B.coreanus*, *B.meridionalis* and *B.chifengi*.

## Introduction

*Bolbelasmus* Boucomont, 1910, one of the largest bolboceratine genera (Li and Wang 2024), was erected by Boucomont (1911) for *Bolbocerasbocchus* Erichson, 1841, *Bolbocerasgallicus* Mulsant, 1842 and *Bolbocerasunicornis* (Schrank von Paula, 1789). The genus *Kolbeus* was also established in the same paper for *Bolbocerasarcuatum* Bates, 1887 and *Bolbocerascoreanum* Kolbe, 1886. *Kolbeus* was later treated as a synonym of the genus *Bolbelasmus* by Cartwright (1953) or a subgenus of the genus *Bolbelasmus* by Nikolajev (1996); the latter classification is followed here.

The genus *Bolbelasmus* currently contains 32 species (including subgenusKolbeus) (Li and Wang 2024). Their distribution is known ranging from the Holarctic and Oriental Regions (Li et al. 2008, Hillert et al. 2016) and the Central America (Moctezuma et al. 2023). Recently, 11 species have been recorded in Asia, of which seven are found in China (*B.coreanus* (Kolbe, 1886); *B.meridionalis* Krikken, 1977; *B.nativusnativus* Krikken, 1977; *B.minutus* Li & Masumoto, 2008; *B.chifengi* Wang & Li, 2024; *B.yutangi* Li & Wang, 2024) (Li and Wang 2024) and the new species described here. Within the genus *Bolbelasmus*, adult males have the following characters: 5.6-15.2 mm body length; clypeus border arcuate, upturned on both sides; the mandibular insertion frequently with a small tubercle; with frontal punctate tubercle; eyes not completely divided by canthus; seven striae between the elytral suture and humeral callus; first elytral stria reaching scutellum; parameres usually weakly sclerotised (Boucomont 1911, Howden 1964, Krikken 1977, Hillert et al. 2016, Zinchenko 2016, Sommer et al. 2021, Li and Wang 2024). The above characteristics can distinguish *Bolbelasmus* from other genera.

Here, a new *Bolbelasmus* species collected in Zhanjiang, Guangdong Province, China, *B.guangdongensis* Liao & Wu sp. nov., is described, illustrated and compared with similar species.

## Materials and methods

The specimens used in this research were collected by hand and at a light trap with a GYZ220-250W E40 mercury blended lamp. Genitalia were macerated in 10% potassium hydroxide (KOH) solution and then rinsed with water (Krikken and Li 2013). A Leica M205 FCA stereomicroscope was used to examine and identify the specimens and capture the specimen images. All images were edited using Leica Application Suite Version 4.12 (Gamma correction, brightness adjustment and contrast adjustment) and Adobe Photoshop 2023 (Background removal, scale bars addition, image stitching and numbering). All the specimens are deposited at the Entomological Museum of Northwest Agriculture and Forestry University, Shanxi Province, China.

## Taxon treatments

### Bolbelasmus (Kolbeus) guangdongensis

Liao & Wu sp. nov.

E39E43E3-6195-56B1-8975-8E82ADE5BF5D

E75064F8-11D7-42EB-BC0D-4EBB487F99E7

#### Materials

- **Type status:** Holotype. **Occurrence:** individualCount: 1; sex: male; lifeStage: adult; preparations: whole animal; disposition: in collection; occurrenceID: 9BB93E1A-84F7-5623-AA5F-03D9508A752A; **Taxon:** kingdom: Animalia; phylum: Arthropoda; class: Insecta; order: Coleoptera; family: Geotrupidae; genus: Bolbelasmus; subgenus: Kolbeus; specificEpithet: *guangdongensis*; taxonRank: species; **Location:** country: China; countryCode: CN; stateProvince: Guangdong; county: Zhanjiang; locality: Huguangyan National Geopark; verbatimElevation: 26 m; verbatimLatitude: 21.13916 N; verbatimLongitude: 110.28635 E; **Identification:** identifiedBy: Liao wei-qi, Wu zheng-wei; **Record Level:** language: en; basisOfRecord: PreservedSpecimen
- **Type status:** Paratype. **Occurrence:** individualCount: 2; sex: 1 female, 1 male; lifeStage: adult; preparations: whole animal; disposition: in collection; occurrenceID: 08BF84F3-2B92-56A9-942C-037EB86557A8; **Taxon:** kingdom: Animalia; phylum: Arthropoda; class: Insecta; order: Coleoptera; family: Geotrupidae; genus: Bolbelasmus; subgenus: Kolbeus; specificEpithet: *guangdongensis*; taxonRank: species; **Location:** country: China; countryCode: CN; stateProvince: Guangdong; county: Zhanjiang; locality: Huguangyan National Geopark; verbatimElevation: 26 m; verbatimLatitude: 21.13916 N; verbatimLongitude: 110.28635 E; **Identification:** identifiedBy: Liao wei-qi, Wu zheng-wei; **Record Level:** language: en; basisOfRecord: PreservedSpecimen

#### Description

**Figure 1.**
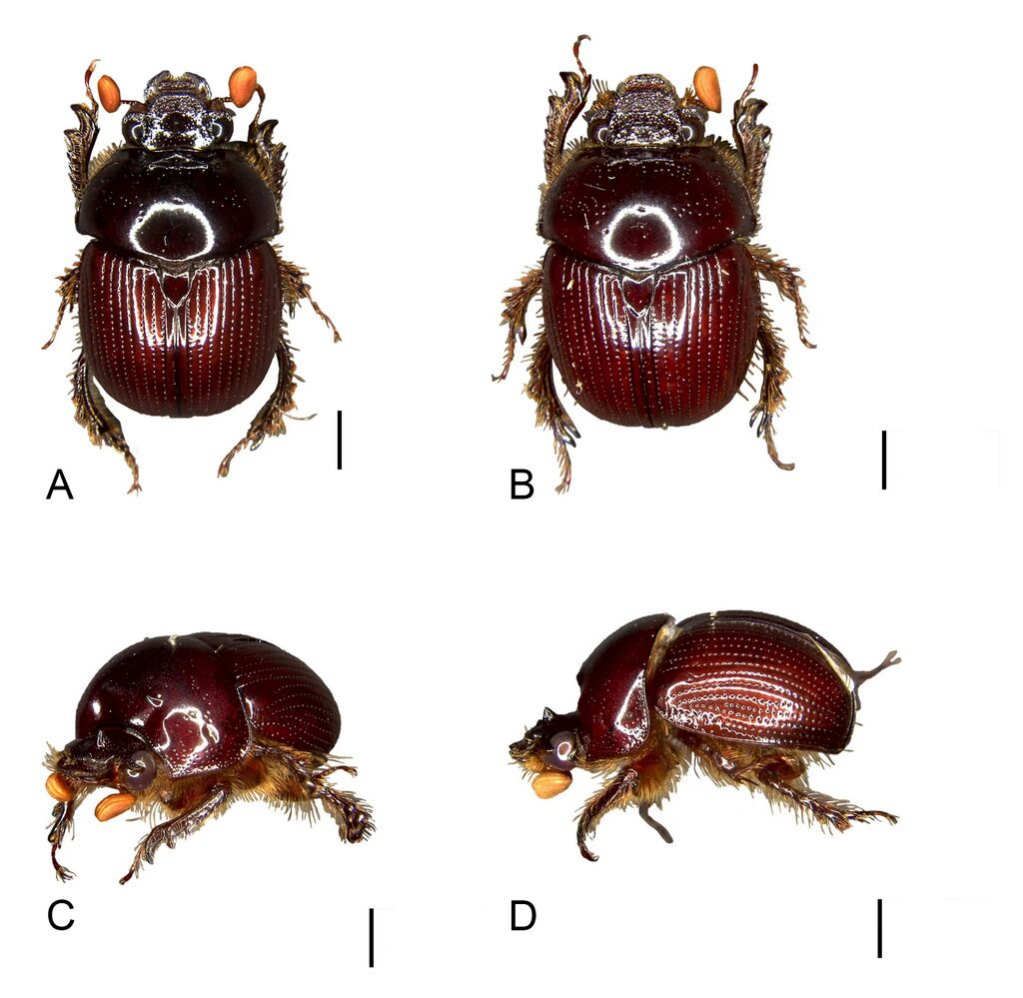
*Bolbelasmusguangdongensis* sp. nov. **A** holotype male, dorsal view; **B** paratype female, dorsal view; **C** holotype, left fronto-lateral view; **D** holotype, left lateral view. Scale bars: 2 mm.

**Figure 2.**
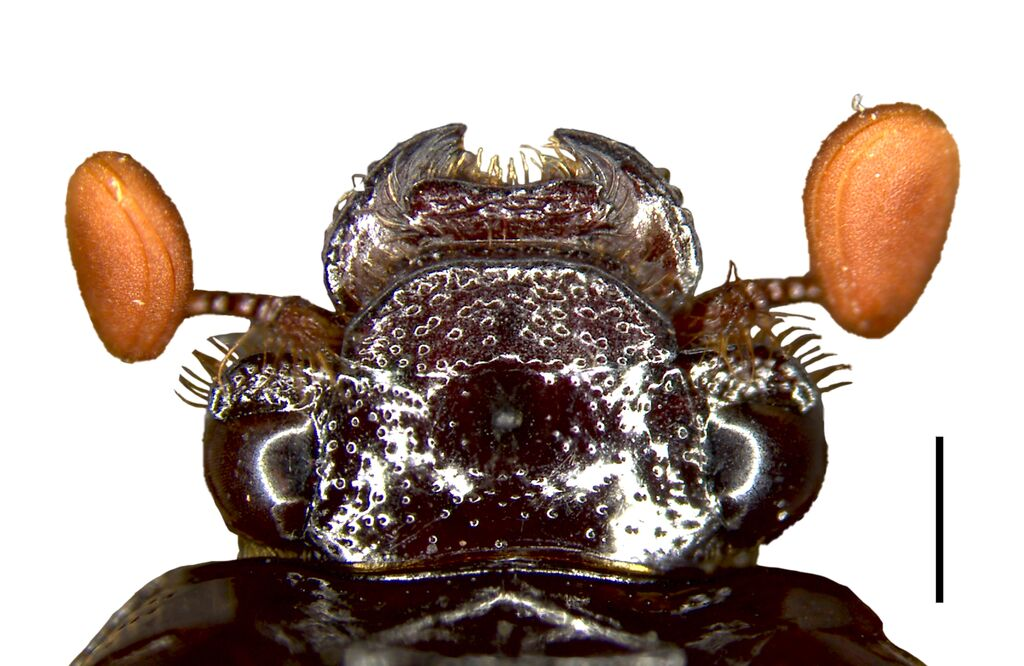
Head of *Bolbelasmusguangdongensis* sp. nov., holotype male, dorsal view. Scale bars: 1 mm.

**Figure 3.**
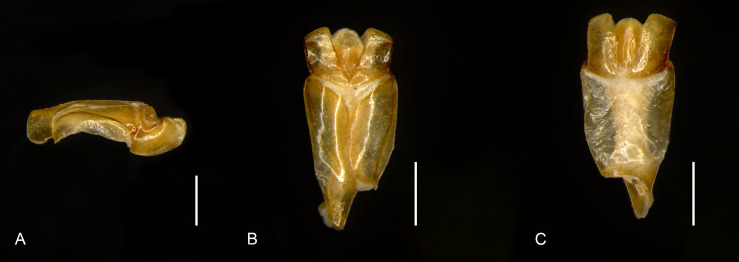
Male genitalia of *Bolbelasmusguangdongensis* sp. nov., holotype. **A** right lateral view; **B** dorsal view; **C** ventral view. Scale bars: 500 μm.

Male: Body length 9.5 mm, maximum width 6.8 mm. Form ovate, sides parallel (Fig. 1A). Colour: dorsum reddish-brown, glossy (Fig. 1A and C-D). *Head*: covered with punctures evenly distributed, except on the mandible (Fig. 2). Labrum slightly convex on both sides of the anterior margin, notched on lateral margins, surface rugulate-punctate (Fig. 2). Clypeus trapezoidal, protrusion at basal angle (Fig. 2). Clypeofrontal suture clear, slightly curved, adjacent to the frontal punctate augmentation. Ocular canthi rounded, encircling the eye incompletely, surface rugulate-punctate (Fig. 2). Antennae form lamellalate, orange (Fig. 2). *Pronotum*: disc maxiumum width approximately 0.7x as long as body length, anteriorly concave, anterolateral pronotal process rounded, inconspicuous (Fig. 1A); posterolateral pronotal process transverse, forming a ridge (Fig. 1A and C). Surface coarsely punctured, banded across the disc, concentrated on the lateral margins, extending towards the middle, gradually thinning out (Fig. 1A and C). Basal fovea conspicuous (Fig. 1C). *Scutellum*: elongate, approximately 0.74x as wide as long, the lateral margins anteriorly are nearly parallel, posteriorly forming a pinched, sharp angle; surface with scattered nearly invisible punctures (Fig. 1A). *Elytron*: semispherical, elytral sutural intervals distinctly convex, thirteen punctate striae distributed on each elytron, uniformly distributed transversely (Fig. 1A and C). *Male genitalia*: small，length 1.2 mm, capsule like (Fig. 3A-C). Phallobase approximately 2.6x as long as paramere; ventral side evenly sclerotised, with two bands, dorsal side weakly sclerotic, membranous (Fig. 3B-C). Parameres diverging from each other, subtrapezoidal in shape; basal expansion and lateral margin strongly sclerotised, curving ventrally in a hook-like fashion (Fig. 3A-C). *Median lobe*: evenly sclerotised, trilobed, ventral apical part with a half-circular opening (Fig. 3B-C).

Female: length 9.7 mm; maximum width 7.7 mm. Similar to the male, except that the frontal punctate augmentation of the head is replaced by a transverse punctate ridge (Fig. 1B).

#### Diagnosis

*Bolbelasmusguangdongensis* sp. nov. belongs to the subgenusKolbeus by pronotal base imarginate (marginate in *Bolbelasmus*) and scutellum elongate (usually short in *Bolbelasmus*) (Krikken 1977). This new species resembles *Bolbelasmuscoreanus*, (Kolbe, 1886) (from Korea and China (Krikken 1977, Li et al. 2008)); *Bolbelasmusmeridionalis* Krikkeni Nikolajev, 1979 (from Indonesia, Thailand, China and Vietnam (Krikken 1977) and *Bolbelasmuschifengi* Wang & Li, 2024 (from China (Li and Wang 2024)) in the body longer, 9 mm (Krikken 1977, Li et al. 2008, Li and Wang 2024) and the frontal tubercle located in junction of clypeofrontal suture (Li and Wang 2024). Their diagnostic characters are summarised in Table 1.

#### Etymology

The new species is named after the place where it was collected.

#### Distribution

Guangdong Province, China.

## Discussion

The flight period of *Bolbelasmusguangdongensis* Liao & Wu sp. nov. adults, recorded in this study, was very short, from late April to early June. The type locality is situated at low altitude, at the shore of a maar in Huguangyan National Geopark. The distribution range of the new species needs further study.

## Supplementary Material

XML Treatment for Bolbelasmus (Kolbeus) guangdongensis

## Figures and Tables

**Table 1.** Diagnostic characters to differentiate B.guangdongensis sp. nov., B.coreanus, B.meridionalis and B.chifengi

	*B.guangdongensis* sp. nov.	*B.coreanus*	*B.meridionalis*	*B.chifengi*
Labrum outer edge	Notched (Fig. 2).	Smooth, widely rounded (fig. 2 in Krikken (1977)).	Smooth, widely rounded (Krikken 1977).	Crenulated (Li and Wang 2024).
Elytral sutural intervals	Distinctly convex (Fig. 1A).	Flat (fig. 28 in Li and Wang (2024)).	Distinctly convex (Li and Wang 2024).	Distinclty convex (Li and Wang 2024).
Parameres	Significantly shorter than the phallobase, diverged, subtrapezoidal shaped, tip rounded (Fig. 3A-C).	Almost as long as phallobase, diverged, irregularly-shaped, tip acute (figs. 11 and 12 in Li et al. (2008)).	Significantly shorter than the phallobase, subparallel, subtrapezoidal-shaped, tip acute (figs. 33 and 34 in Li and Wang (2024)).	Almost as long as phallobase, diverged, irregularly-shaped, tip rounded (figs. 31 and 32 in Li and Wang (2024)).
Medium lobe	Elongate, tip tapered, nearly level with the apex of parameres (Fig. 3B-C).	Stout, covered in parameres (figs. 11 and 12 in Li et al. (2008)).	Covered by parameres (figs. 33 and 34 in Li and Wang (2024)).	Elongate; broad at the tip, nearly level with the apex of the parameres (figs. 31 and 32 in Li and Wang (2024)).
